# MicroRNA–mRNA Pairs Associated with Outcome in AML: From *In Vitro* Cell-Based Studies to AML Patients

**DOI:** 10.3389/fphar.2015.00324

**Published:** 2016-01-28

**Authors:** Neha S. Bhise, Lata Chauhan, Miyoung Shin, Xueyuan Cao, Stanley Pounds, Vishal Lamba, Jatinder K. Lamba

**Affiliations:** ^1^Department of Pharmacotherapy and Translational Research, University of FloridaGainesville, FL, USA; ^2^Department of Experimental and Clinical Pharmacology, University of MinnesotaMinneapolis, MN, USA; ^3^Department of Biostatistics, St. Jude Children’s Research HospitalMemphis, TN, USA

**Keywords:** miRNA, microRNA, cytarabine, acute myeloid leukemia, gene expression

## Abstract

Cytarabine is the primary chemotherapeutic agent used for treatment of acute myeloid leukemia (AML). Disease relapse after initial remission remains one of the most pressing therapeutic challenges in the treatment of AML. Relapsed disease is often resistant to cytarabine and subsequent salvage therapy is ineffective. Recent studies have shown that some microRNAs (miRNAs) are associated with prognosis, but have not yet explored the role of miRNAs in cellular response to cytarabine. We identified 20 miRNAs that associate with the *in vitro* cytarabine chemo-sensitivity or apoptotic response of eight AML cell lines. Out of the 20 miRNAs, data on 18 miRNAs was available in AML patients from The Cancer Genome Atlas database. Our stepwise-integrated analyses (step 1 – miRNA–target mRNA that were significantly correlated in AML patients; step 2 – mRNAs from step 1 with significant association with overall survival (OS)) identified 23 unique miRNA–mRNA pairs predictive of OS in AML patients. As expected HOX genes (HOXA9, HOXB7, and HOXA10) were identified to be regulated by miRs as well as predictive of worse OS. Additionally, miR107-Myb, miR-378-granzyme B involved in granzyme signaling and miR10a-MAP4K4 were identified to be predictive of outcome through integrated analysis. Although additional functional validations to establish clinical/pharmacologic importance of miRNA–mRNA pairs are needed, our results from RNA electrophoretic mobility shift assay confirmed binding of miR-10a, miR-378, and miR-107 with their target genes GALNT1, GZMB, and MYB, respectively. Integration of pathogenic and pharmacologically significant miRNAs and miRNA–mRNA relationships identified in our study opens up opportunities for development of targeted/miRNA-directed therapies.

## Introduction

Acute myeloid leukemia (AML) is a hematological malignancy characterized by the presence of immature abnormal myeloid cells in bone marrow. It is a heterogeneous disease with various subtypes classified based on the morphology, immunophenotype, and cytogenetics that are associated with outcome (Döhner et al., 2010). In spite of advances in recent years, 5-year overall survival (OS) is roughly 60% for children and ∼25% for adults (Cancer Facts and Figures, American Cancer Society). Cytarabine (1-β-arabinofuranosylcytosine, ara-C), a nucleoside analog, is the most widely used chemotherapeutic agent used in combination with an anthracycline for treatment of AML. Cytarabine requires intracellular activation to form an active triphosphate metabolite that triggers apoptosis by inhibiting DNA synthesis. Although chemotherapeutic regimens including cytarabine induce complete response in 65–80% of AML patients, the majority of these patients suffer from disease relapse within 2 years of diagnosis (Cros et al., 2004). This can be partly attributed to the development of resistance of leukemic cells to cytarabine-based chemotherapy regimens (Montillo et al., 1998; Estey, 2000). Several factors such as molecular and cytogenetic subtype, differential gene-expression profiles, and epigenetics can account for development of resistance in AML (Sasaki et al., 2008; Peters et al., 2010; Mitra et al., 2011; Cao et al., 2013; Gamazon et al., 2013; Mortland et al., 2013). We have previously identified gene expression signatures in AML patients predicting beneficial and detrimental patterns associated with cytarabine-based response (Lamba et al., 2011). Others have identified gene-expression differences between cytarabine-sensitive and -resistant cell lines in order to understand the molecular mechanisms underlying cytarabine resistance (Abe et al., 2006; Song et al., 2009).

MicroRNAs (miRNAs, miRs) are non-coding RNAs of 22–25 nucleotides that regulate gene expression. miRNAs bind to the complimentary sequence of messenger RNAs (mRNAs). In many cases, this binding suppresses mRNA translation or promotes mRNA degradation, thereby reducing expression at the protein level (Bartel, 2004). It has been shown that miRNAs play an important role in various cancers by regulating genes involved in cell proliferation, differentiation, and apoptosis (Zhang et al., 2010; Paik et al., 2011; Chen et al., 2013; Palma et al., 2014). Likewise, several recent studies have identified various miRNAs that differentiate the disease subgroups, associate with disease development, and associate with clinical prognosis of AML (Dixon-McIver et al., 2008; Jongen-Lavrencic et al., 2008; Marcucci et al., 2011; Starczynowski et al., 2011; Li et al., 2013; Jinlong et al., 2015; Shibayama et al., 2015). However, these valuable contributions have not yet carefully examined the roles of miRNAs in the cellular response of AML to cytarabine. Therefore, in this study, we characterized the association of miRNA expression with apoptotic response to cytarabine to identify candidate miRNAs for further evaluation of their relationship with mRNAs and OS in The Cancer Genome Atlas (TCGA) cohort of 200 AML patients.

## Materials and Methods

**FIGURE 1:**
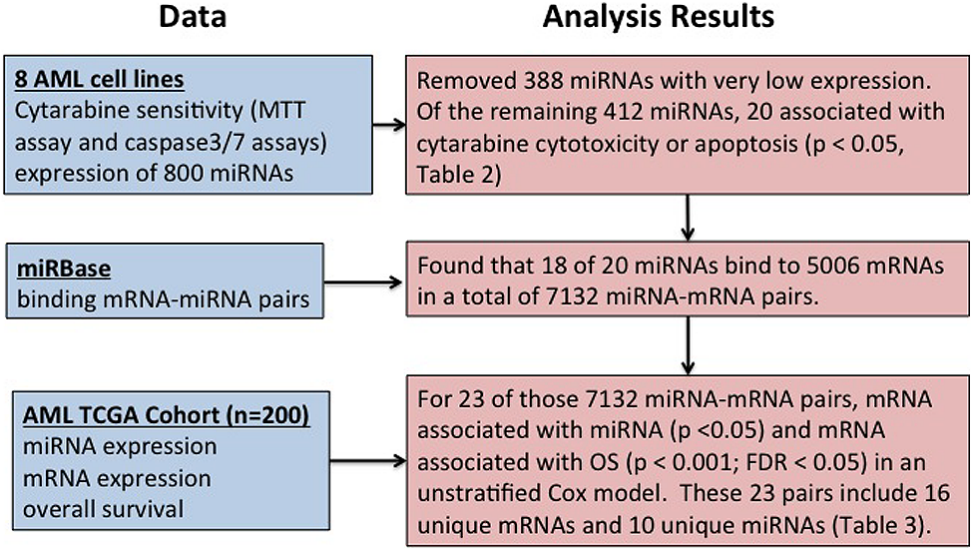
**Overall summary of the study.** Study design for identification of microRNAs (miRNA) influencing cytarabine chemo-sensitivity and survival in acute myeloid leukemia (AML) patients.

**Figure 1** shows overall study design.

### Cell Culture and Regents

The AML cell lines HL-60, MV-4-11, Kasumi-1, THP-1, AML-193, and KG-1 were obtained from ATCC (Manassas, VA, USA), while ME-1 and MOLM-16 cell lines were obtained from DSMZ (Braunschweig, Germany). All the cell lines were cultured in the media as recommended by the supplier and were maintained in a humidified incubator at 37°C with 5% CO_2_. The cells were passaged every 2–3 days in order to maintain them in logarithmic growth phase. Cytarabine and the 3-(4,5-dimethylthiazol-2-yl)-2,5-diphenyltetrazolium bromide (MTT) reagent were purchased from Sigma–Aldrich (St. Louis, MO, USA). Stock concentrations for cytarabine (5 mg/ml) were prepared in sterile water and stored in –20°C in aliquots.

### Cytotoxicity Assay

Cytarabine cytotoxicity was determined using the MTT assay. Briefly, AML cell lines were plated in 96-well plate at seeding density of 2.5 × 10^5^ cells/ml and incubated at 37°C overnight. After 24 h of recovery time, the cells were exposed to varying concentrations (200, 100, 50, 5, 0.5, 0.01, and 0 μM) of cytarabine. Cell viability was determined 48 h post cytarabine treatment by incubation with MTT reagent followed by measuring the absorbance at 570 nm using Synergy plate reader (BioTek, USA). The percent cell survival at each concentration was calculated using the Gen5^TM^ Software version 1.11 (Winooski, VT, USA). The area under the survival curve (AUC) was calculated by the trapezoidal method using the GraphPad Prism software version 6 (La Jolla, CA, USA).

### Apoptosis Assay

The apoptotic activity of AML cell lines following treatment (48 h) with varying concentration of cytarabine (as indicated above) was determined using the Caspase-Glo^^®^^ 3/7 assay as per manufacturer’s instructions (Promega, USA). Forty-eight hours of post cytarabine treatment, luminescence was read using Synergy plate reader (BioTek, USA). The luminescence produced is directly proportional to the caspase activity. The caspase activity at each concentration was normalized to the control and the area under the relative caspase activity curve (AUC) was calculated by the trapezoidal method using the GraphPad Prism software version 6 (La Jolla, CA, USA).

### MicroRNA Expression Analysis

For determination of miRNA expression, total RNA was isolated using mirVana^TM^ miRNA Isolation kit (Life Technologies, USA) as per the manufacturer’s protocol. The RNA quality and concentration was measured using NanoDrop 2000 UV-Vis spectrophotometer (Thermo Scientific, USA). A total of 100 ng of purified total RNA was assayed for determination of 800 human miRNA expression using the nCounter Human v2 miRNA Expression Assay kit (Nanostring Technologies, USA). miRNA expression data normalization was performed using the nSolver^TM^ Analysis Software (Nanostring Technologies) according to the manufacturer’s instructions. In order to avoid using the miRNAs with a very low expression, we further filtered out the miRNAs with expression counts <30 (two times the mean ± 2*SD* of negative control value, accounting for the background noise). Total 412 miRNAs with expression counts >30 were evaluated for differential expression between sensitive and resistant cell lines and for their correlation with cytarabine chemosensitivity.

### TCGA Data

The clinical outcome data, mRNA expression and miRNA expression data for AML patients were extracted from The Cancer Genome Atlas (TCGA) Data Portal^1^ (Cancer Genome Atlas Research Network, 2013). Out of the 200 AML patients in TCGA database, 197 patients had gene expression profiling data available and 187 patients had miRNA expression data available. One hundred and eighty-six patients had both gene expression and miRNA expression data available. Out of the 186 AML patients, 13 patients lacked valid survival information and two patients lacked cytogenetically defined risk information. Thus, data for a total of 186 patients were used to evaluate miRNA–mRNA associations, 173 patients used to evaluate mRNA-OS associations, and miRNA-OS associations (171 for stratified analyses).

### Statistical Analysis

For each miRNA, Spearman’s rank-based correlation was used to measure the association with cytarabine treatment response or apoptosis in the eight cell lines. For each miRNA–mRNA pair with predicted binding sites defined by miRBase^2^ (release 21), Spearman’s rank-based correlation was used to evaluate the association of miRNA expression with mRNA expression on the TCGA AML cohort. The *p*-value for the Spearman statistic was determined by 10,000 permutations. For each miRNA or mRNA, Cox regression [or Jung’s statistic] was used to evaluate the association of expression with OS. FDR was estimated by Pounds and Cheng’s (2006) robust FDR method.

### Electrophoretic Mobility Shift Assays

The functional validation for binding efficiencies between selected miRNAs and mRNAs was performed using the electrophoretic mobility shift assays (EMSAs) as described previously (Yu et al., 2015). The binding free energy between the respective mRNA and miRNA pair (demonstrating inverse relationship) was predicted using the RNAhybrid software. The miRNA oligonucleotides were labeled with cy5^TM^ dye on their 5′ ends. The 2′ *O*-methyl-modified mRNA oligonucleotides were labeled with IRDye^^®^^ 800 (LI-COR Biosciences, USA) dye on their 5′ ends. The labeled oligonucleotides were synthesized by Integrated DNA Technologies (Coralville, IA, USA). RNA EMSA experiment was performed using the LightShift Chemiluminescent RNA EMSA Kit (Thermo Scientific, USA) according to the manufacturer’s protocol. The mRNA oligonucleotide was heated for 10 min at 80°C and then placed on ice in order to relax the secondary structures. In each 20 μl binding reaction, 200 nM miRNA oligonucleotide and/or mRNA oligonucleotide were mixed with RNA EMSA binding buffer and incubated at 25°C for 25 min. The reaction mixtures were separated on a 15% polyacrylamide gel by electrophoresis at 4°C. The binding reactions were electrophoretically transferred onto nylon membrane and the resulting mobility shifts were imaged using Odyssey CLx Infrared System (LI-COR Biosciences, USA).

## Results

### Cytarabine Chemo-Sensitivity of AML Cell Lines

The AML cell lines showed considerable variability in cytarabine sensitivity as measured by cytotoxicity AUC and apoptosis AUC in the MTT assay (**Table 1**; Supplementary Figures S1A–H). Based on the cytotoxicity AUC, HL-60, MV-4-11, KG-1, and ME-1 were classified as sensitive (cytarabine AUC < 12000), while MOLM-16, AML-193, Kasumi-1, and THP-1 were classified as resistant cell lines (cytarabine AUC > 12000).

**Table 1:** Characterization of acute myeloid leukemia (AML) cell lines for cytarabine chemosensitivity.

Cell lines	Cytogenetics/molecular abnormality	Ara-C cytotoxicity AUC (±*SD*)	Ara-C apoptosis AUC (±*SD*)
Kasumi-1	*t*(8;21)(q22;q22) → RUNX1/AML1-RUNX1T1/ETO fusion gene; TP53 mutant gene	14713 (±582)	409.3 (±20)
THP-1	*t*(9;11)(p21;q23) → MLL-AF9 fusion gene; CDKN2A, KDM6A, NRAS mutant genes	17170 (±1341)	1544.5 (±2)
MOLM-16	*t*(6;8)(q21;q24.3) and t(9;18)(q13;q21)	12021 (±480)	490.2 (±18)
AML-193	+der(17)t(17;17)(p13.1;q21.3)	12988 (±366)	852.4 (±34)
MV-4-11	FLT3 ITD mutation, t(4;11)(q21;q23) → MLL-AF4 fusion gene	5011 (±442)	458.4 (±22)
ME-1	inv(16)(p13q22) → CBFB-MYH11 fusion gene	6497 (±280)	753.6 (±33)
KG-1	NRAS mutation, P53 mutation, RB1 rearrangement	5939 (±464)	472.5 (±16)
HL-60	CDKN2A, NRAS, TP53 mutant genes	4597 (±397)	555.6 (±29)

### MicroRNAs Associated with Cytarabine Chemo-Sensitivity in AML Cell Lines

Of the 800 human miRNAs quantitated using the nCounter Nanostring platform we excluded 388 miRNAs due to very low expression and 412 miRNA were analyzed further. Twenty miRNAs were associated with cellular viability (nine miRs) or caspase activation (11 miRs) in AML cell lines post treatment with cytarabine (**Table 2**). Expression of miR-25-3p, miR-148b-3p, miR-107, miR-374-5p, miR-425-5p were positively associated with AUC for cell survival post-cytarabine treatment and miR-16-5p, miR-24-3p, miR-196a-5p, and miR-155-5p were negatively associated with AUC for cell survival post-cytarabine treatment (*p* < 0.05). (Selected miRNAs are shown in Supplementary Figure S2.) Expression levels of miR-10a-5p, miR-29a/b-3p, miR-30e-5p, miR-33a-5p, miR-378a/g were positively and expression levels miR-197-3p, miR-27b-3p, miR-324-5p, and miR-421 were negatively associated with AUC for caspase-3/7 activation (apoptosis) post cytarabine treatment (**Table 2**, *p* < 0.05). Using Ingenuity pathway analysis tool, the miRNAs that were correlated with cytarabine chemosensitivity were also found to potentially impact important biological process relevant to leukemia/cancer (Supplementary Figure S3).

**Table 2:** MicroRNAs significantly associated with cytarabine-induced cytotoxicity AUC and cytarabine-induced apoptosis (caspase-3/7 activity).

MicroRNA	Spearman *r*	*p*-value
**Cytarabine-induced cell cytotoxicity AUC**		
hsa-miR-107	0.7619	0.028
hsa-miR-148b-3p	0.7381	0.037
hsa-miR-155-5p	-0.8095	0.015
hsa-miR-16-5p	-0.7381	0.037
hsa-miR-196a-5p	-0.7857	0.021
hsa-miR-24-3p	-0.8095	0.015
hsa-miR-25-3p	0.7857	0.021
hsa-miR-374a-5p	0.7381	0.037
hsa-miR-425-5p	0.7619	0.028
**Cytarabine-induced apoptosis AUC**		
hsa-miR-10a-5p	0.7857	0.021
hsa-miR-197-3p	-0.8571	0.007
hsa-miR-27b-3p	-0.7186	0.045
hsa-miR-29a-3p	0.881	0.004
hsa-miR-29b-3p	0.7857	0.021
hsa-miR-30e-5p	0.7381	0.037
hsa-miR-324-5p	-0.9048	0.002
hsa-miR-33a-5p	0.8095	0.015
hsa-miR-378a-3p	0.8095	0.015
hsa-miR-378g	0.7381	0.037
hsa-miR-421	-0.7619	0.028

### Pairs of Significantly Correlated mRNAs and miRNAs that Associate with Overall Survival of AML Patients

Of 20 miRNAs identified above, data on 18 were available in AML patients from TCGA database and were tested for associations with risk group and outcome. As shown in Supplementary Table S1, seven of these miRNAs (miR-10a, miR-16, miR-196a, miR-197, miR-421, miR-155, and miR24) demonstrated significant difference in expression levels among the three risk groups. In risk stratified analysis miR107, miR-155, miR-196a, miR-25, and miR29b were associated with worse OS, whereas miR-25 was predictive of better OS in AML patients at *p* < 0.05.

Using miRBase^2^ (release 21), we determined that 5006 probe sets representing 2830 gene with binding sites for these 18 miRNAs. These 5006 mRNAs and 18 miRNAs belong to 7132 distinct miRNA–mRNA pairs. Using the analysis strategy outlined in **Figure 1**, we found that 23 of the 7132 miRNA–mRNA pair’s satisfied criteria listed below (**Table 3**):

(a) significant association between miRNA and target mRNA (*p* < 0.05; 1532 pairs) and
(b) mRNA expression associated significantly with OS in an unstratified Cox regression model (*p* < 0.001; *FDR* < 0.05).

**Table 3:** miRNA–mRNA pairs predictive of overall survival in AML Patients (data from TCGA).

miRNA–mRNA Pair				miRNA–mRNA correlation		mRNA-risk group		mRNA-OS		
				Spearman Correlation		Kruskal–Wallis		Unstratified Cox model		
miRNA	mRNA probe	mRNA gene	mRNA Chr	*r*	*p*-value	*p*-value	*FDR*	*HR*	*p*-value	*FDR*
mir-107	212202_s_at	TMEM87A	chr15q15.1	-0.208	**0.004**	**0.000**	0.001	0.386	**0.000**	0.045
mir-107	204798_at	MYB	chr6q23.3	-0.235	**0.001**	**0.050**	0.075	0.430	**0.000**	0.004
mir-10a	201724_s_at	GALNT1	chr18q12.2	-0.418	**0.000**	**0.000**	0.000	0.327	**0.000**	0.006
mir-10a	218181_s_at	MAP4K4	chr2q11.2	-0.445	**0.000**	**0.046**	0.072	0.558	**0.001**	0.045
mir-10a	222273_at	PAPOLG	chr2p16.1	-0.174	**0.018**	0.429	0.350	0.392	**0.000**	0.013
mir-148b	218181_s_at	MAP4K4	chr2q11.2	0.240	**0.001**	**0.046**	0.072	0.558	**0.001**	0.045
mir-16	213150_at	HOXA10^∗^	chr7p15.2	0.441	**0.000**	**0.000**	0.000	1.216	**0.000**	0.041
mir-16	201724_s_at	GALNT1	chr18q12.2	-0.217	**0.003**	**0.000**	0.000	0.327	**0.000**	0.006
mir-16	212314_at	SEL1L3	chr4p15.2	0.263	**0.000**	**0.000**	0.000	1.446	**0.001**	0.046
mir-16	209193_at	PIM1	chr6p21.2	0.247	**0.001**	**0.002**	0.006	1.713	**0.000**	0.045
mir-196a	214651_s_at	HOXA9^∗^	chr7p15.2	0.293	**0.000**	**0.000**	0.000	1.166	**0.001**	0.046
mir-196a	204779_s_at	HOXB7	chr17q21.32	0.223	**0.002**	**0.000**	0.000	1.276	**0.001**	0.046
mir-196a	201852_x_at	COL3A1	chr2q32.2	0.252	**0.001**	0.152	0.170	0.783	**0.000**	0.045
mir-196a	213687_s_at	RPL35A	chr 3q29	-0.174	**0.018**	0.300	0.276	0.399	**0.000**	0.042
mir-197	212202_s_at	TMEM87A	chr15q15.1	-0.184	**0.012**	**0.000**	0.001	0.386	**0.000**	0.045
mir-197	218181_s_at	MAP4K4	chr2q11.2	0.159	**0.030**	**0.046**	0.072	0.558	**0.001**	0.045
mir-29b	207106_s_at	LTK^∗^	chr15q15.1	-0.184	**0.012**	**0.000**	0.000	0.790	**0.001**	0.046
mir-29b	56919_at	WDR48	chr3p22.2	-0.169	**0.021**	0.068	0.095	0.514	**0.000**	0.009
mir-30e	204779_s_at	HOXB7	chr17q21.32	-0.325	**0.000**	**0.000**	0.000	1.276	**0.001**	0.046
mir-30e	201724_s_at	GALNT1	chr18q12.2	0.208	**0.004**	**0.000**	0.000	0.327	**0.000**	0.006
mir-30e	218313_s_at	GALNT7	chr4q34.1	0.329	**0.000**	0.106	0.132	0.417	**0.000**	0.024
mir-378	210164_at	GZMB	chr14q12	-0.205	**0.005**	0.199	0.207	1.439	**0.001**	0.046
mir-421	214651_s_at	HOXA9^∗^	chr7p15.2	-0.277	**0.000**	**0.000**	0.000	1.166	**0.001**	0.046

*^∗^Except for HOXA9, HOXA10, and LTK, all other genes were also significantly associated with OS (p < 0.05) in risk stratified analysis. FDR was estimated by Pounds and Cheng (2006) robust FDR method.*

*Bold indicates significance for p-value with p < 0.05 (Spearman Correlation and Kruskal–Wallis) and p < 0.001 (Unstratified Cox model).*

These 23 pairs included 16 unique genes and 10 unique miRNAs (**Table 3** – some mRNAs and some miRNAs belonged to multiple pairs). A positive correlation of mRNA and miRNA was observed for 10 of these pairs and a negative correlation was observed for the other 13 pairs.

**FIGURE 2:**
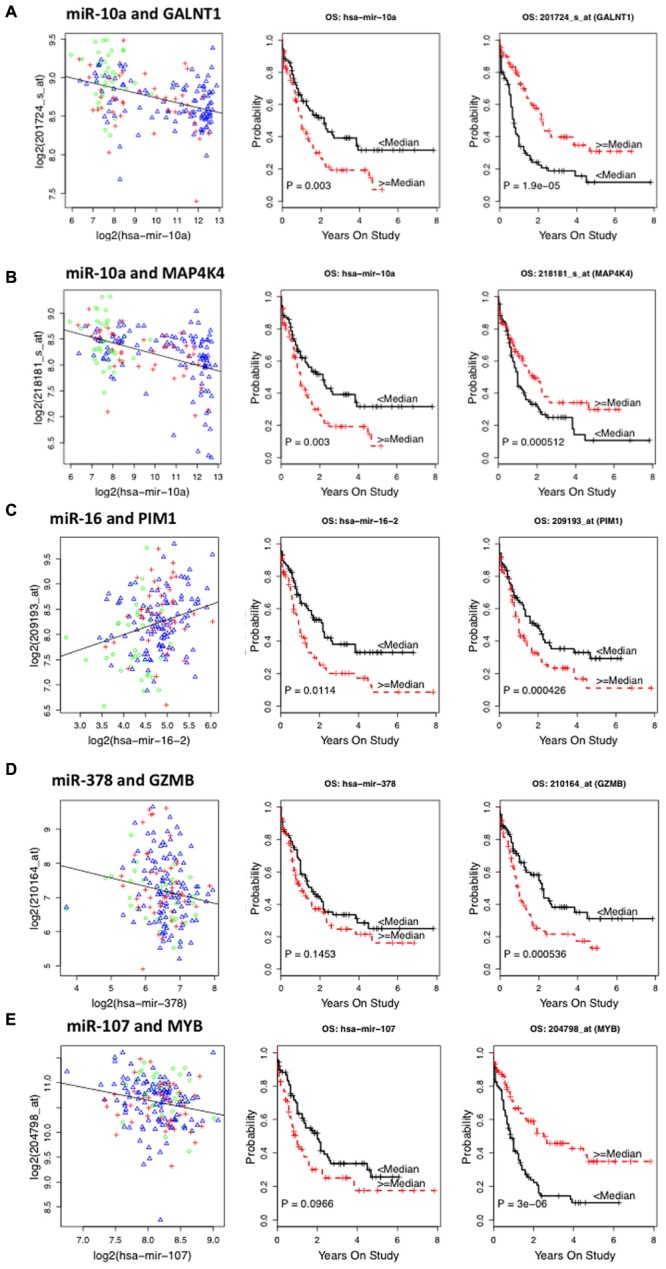
**Representative plots showing correlation between miRNA–target messenger RNA (mRNA) and survival curves of miRNA and mRNA expression with overall survival (OS) in AML patients from The Cancer Genome Atlas (TCGA) database. (A)** Correlation plot of miR10a–GALNT1 mRNA levels and Kaplan–Meier survival curves of miR10a and GALNT1 expression with OS. **(B)** Plots for miR-10a–MAP4K4 pair. **(C)** Plots for miR-16 and Pim1 pair. **(D)** Plots for miR-378 and GZMB pair. **(E)** Plots for miR-107 and Myb pair.

Among mRNA-OS associations COL3A1, GALNT1, GALNT7, LTK, MAP4K4, MYB, PAPOLG, RPL35A, TMEM87A, and WDR48 were associated with better OS and HOX family genes (HOXA9, HOXA10, HOXB7), GZMB, SE1L3, and an oncogene PIM1 were associated with inferior outcome (**Table 3**). Since nine of these genes demonstrated significant association with risk groups we also performed risk-stratified analysis and all but three genes HOXA9, HOXA10, and LTK were significantly associated with OS in risk stratified analysis, indicating that for these genes the observed association with OS might be driven by risk group characteristics. **Figure 2** shows the representative correlation plots as well as overall curves for miR10a-GALNT1, miR10a-MAP4K4, miR16-Pim1, miR378-GZMB, and miR107-MYB.

**FIGURE 3:**
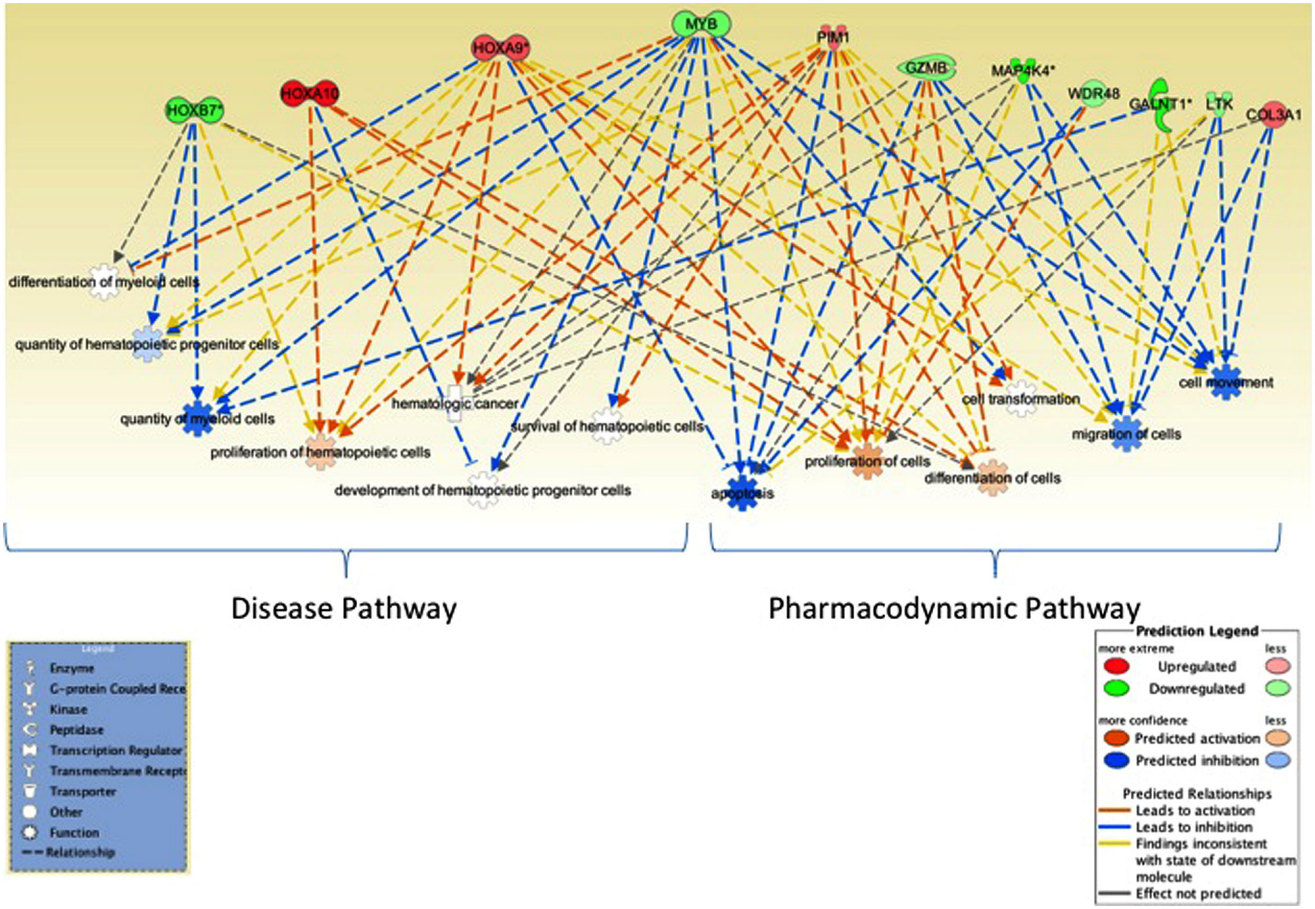
**Network analysis.** Ingenuity pathway analysis tool was utilized to evaluate the genes identified in miRNA–mRNA-OS analysis. Several genes mapped to biological processes of relevance to hematological malignancies, myeloid leukemia as well as to cell proliferation and apoptosis, RNA expression. Genes associated with better OS are in green and ones with worse OS are in red.

We further utilized Ingenuity pathway analysis tool to map the 16 unique genes identified in integrative analysis and as shown in **Figure 3**, these genes were associated with cell proliferation, apoptosis, RNA expression, and quantity of hematopoietic progenitor cells, hematological cancer and myeloid leukemia.

### Functional Validation Using RNA Electrophoretic Mobility Shift Assays (RNA EMSA)

**FIGURE 4:**
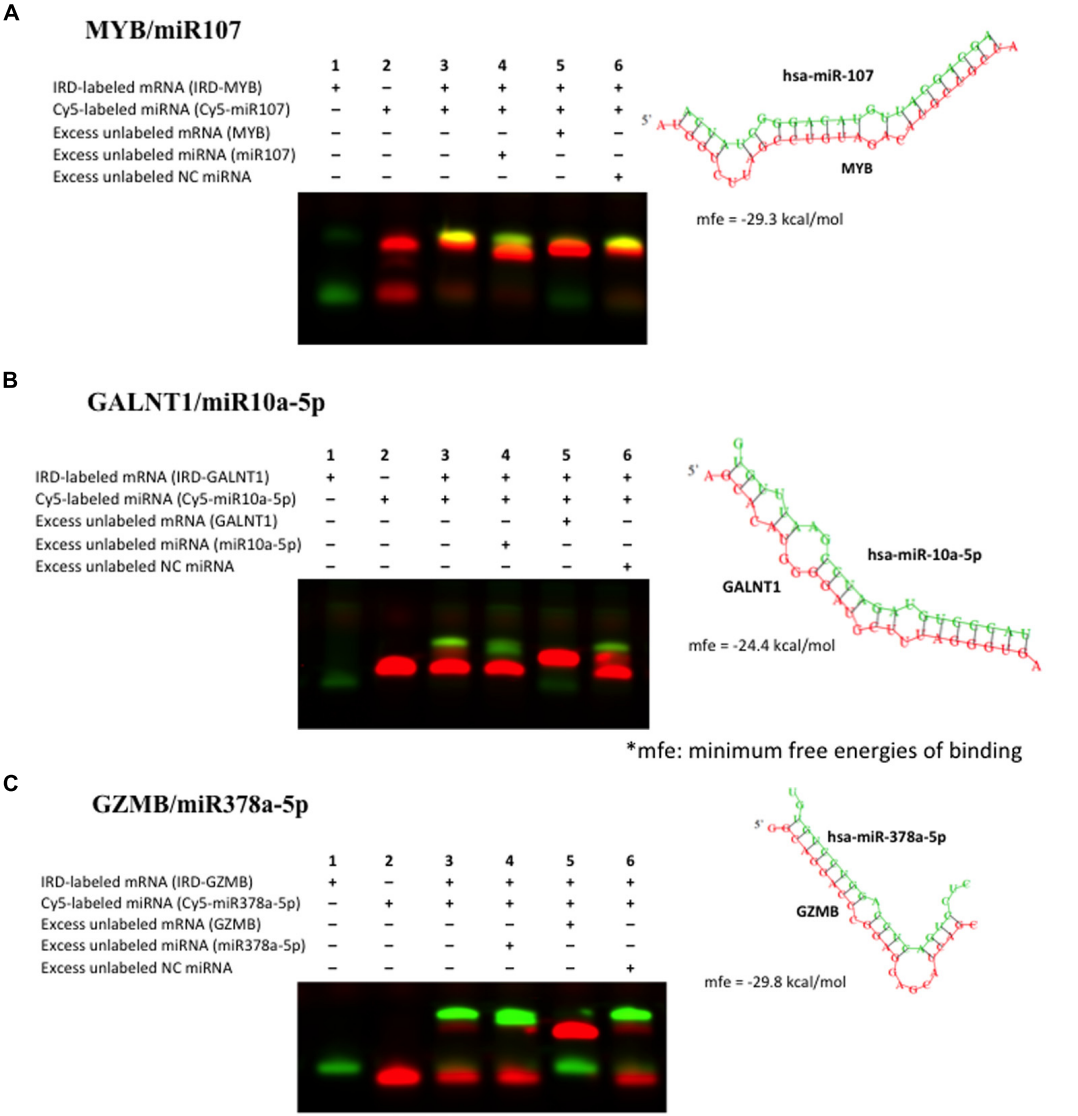
**Validation of binding interaction between miRNA–mRNA by RNA electrophoretic mobility shift assays (RNA EMSAs): (A) miR107–MYB; (B) miR10a–GALNT1 and (C) miR378–GZMB.** RNA EMSAs were performed using cy5-labeled respective miRNA oligonucleotide and 2′-*O*-methyl modified and IRD-800 labeled mRNA oligonucleotides. Lanes 1 and 2 show the mobility of the respective labeled mRNA or miRNA oligonucleotide. Lane 3 shows the mobility of the labeled miRNA oligonucleotide with its target mRNA oligonucleotide. Lanes 4 and 6 show the mobility of labeled mRNA oligonucleotide in presence of excess unlabeled specific competitor miRNA oligonucleotide and excess unlabeled non-specific competitor (NC). Lane 5 shows the mobility of labeled mRNA oligonucleotide in presence of excess unlabeled specific competitor mRNA oligonucleotide.

To further validate the miRNA–mRNA pairs discovered as above, we performed RNA EMSAs. We first selected miRNA–mRNA pairs from **Table 3** that demonstrated significant inverse relationships. Binding free energy for these miRNA–mRNA pairs was calculated using RNAhybrid software. The 3′UTR sequence of mRNA was obtained from the UCSC Genome browser and miRNA sequence was obtained from miRBase software. Of miRNA–mRNAs demonstrating minimum free energy of binding <–24 kcal/mol miR107-MYB; miR378a-GZMB, and miR10a-GALNT1 were validated by RNA EMSAs. EMSA results confirmed binding for miR-107-MYB, miR-10-GALNT1, and miR-378-GZMB and are shown in **Figure 4**, respectively. As shown in lane 3 in **Figure 4**, the EMSA results for miR-107, miR-10a, and miR-378 demonstrate binding of this miRNA with their respective mRNA target sequences confirming the thermodynamic stability of these complexes predicted in the *in silico* analysis. In addition, we observed competition of binding in mRNA–miRNA complexes after adding excess unlabeled specific miRNA probe (**Figure 4**, lane 4), excess unlabeled mRNA probe (**Figure 4**, lane 5) but not by adding excess unlabeled non-specific probe (**Figure 4**, lane 6).

## Discussion

Acute myeloid leukemia is a heterogeneous disease with dismal outcome. Additional complexity is added by very heterogeneous nature of AML with cytogenetic abnormalities used for risk classification in AML patients. Although cytarabine has been the backbone of AML chemotherapy for more than 50 years, there are still gaps in our understanding of the molecular mechanisms contributing to development of drug resistance in AML. Although advances in supportive care have improved, the treatment strategies have not changed much with cytarabine being still the main player. Thus, understanding the molecular mechanisms underlying cytarabine resistance will be of great interest in developing predictive models of outcome as well as for developing novel therapeutic strategies. Recent research has shown the significant role of miRNAs in normal hematopoiesis (Chen et al., 2004; Garzon and Croce, 2008; O’Connell et al., 2008) as well as miRNA deregulation in AML. miRNAs (miR-125, miR-146, miR-142, miR-155, miR-29, miR-181, let-7a, etc.) of potential prognostic significance have been identified (Khalaj et al., 2014). Recent data supports putative diagnostic role of miR-155 in all AML subgroups and miR-196b within M4-5 subgroups (Yan et al., 2015). miR-9 has been also implicated in promoting proliferation of leukemic cells in normal karyotype AML by targeting Hes1 (Tian et al., 2015). However, the role of miRNAs in development of resistance to cytarabine and thus inferior clinical outcome has not been investigated in detail. In this study, we report results of our genome-wide evaluation to identify miRNAs associated with cytarabine sensitivity in eight AML cell lines as well as their impact on clinical outcome in AML patients (**Figure 1** outlines overall study design and results).

We screened expression levels of 800 human miRNAs in eight AML cell lines and after filtering out miRNAs with very low expression, evaluated 412 miRNAs for association with cytarabine chemo-sensitivity, measured as cell viability and apoptosis induction following cytarabine treatment. Twenty unique miRNAs were predictive of cytarabine chemo-sensitivity in AML cell lines, and 18 of these were further evaluated in AML patients from TCGA database. Seven of these were differentially expressed among AML risk groups (favorable, intermediate and poor; *p* < 0.05) and after risk stratification five miRNAs (miR-107, miR-155, miR-25, miR-29b, and miR-196a) were associated with OS (Supplementary Table S1). All but miR-25 were associated with worse OS in AML patients.

Among the miRNAs associated with OS, miR-155 is located in a non-coding RNA transcript cluster called B-cell integration cluster (BIC), which has been shown to cooperate with c-Myc (Clurman and Hayward, 1989; Tam, 2001). miR-155 is considered as an oncomiR with implications in pathogenesis of AML (O’Connell et al., 2008; Palma et al., 2014); it has been associated with SHIP1 (negatively regulator of PI3K/AKT pathway) and CEBP-β (Gorgoni et al., 2002; O’Connell et al., 2009). Our results show that miR-155 is associated with cytarabine sensitivity which is not in consensus with its association with inferior outcome, thereby indicating that miR-155 (which is also differentially expressed among risk groups) might have significant impact on disease pathogenesis but might not be impacting drug response. miR-29 family members are regulators of myeloid differentiation and have been shown to be deregulated in AML (Garzon et al., 2008, 2009a; Han et al., 2010). MiR29a/29b have been associated with expression levels of oncogenes MCL1, CDK6, IGFR, and JAK2 (Mott et al., 2007; Garzon et al., 2009a) as well as have been shown to target DNA modifying genes DNMTs and TET2 (Garzon et al., 2009b; Cheng et al., 2013).

In step-wise integrated analysis we identified 23 miRNA–mRNA pairs predictive of survival in AML patients and these pairs were mapped to 16 unique mRNAs.

We further validated miR107-MYB, miR-10a-GALNT1, and miR378-GZMB miRNA–mRNA pairs using electrophoretic mobility shift assays (**Figure 4**), which confirmed the binding of these oligos as supported by *in silico* analysis indicating miRNAs in regulating gene expression of these target genes by binding to specific seed sequences.

As expected HOX genes (HOXA9, HOXA10, and HOXB7) were predictive of worse outcome. miR-196a-1 which was associated cytarabine *in vitro* sensitivity in AML cell lines was positively correlated in expression with HOXA9 and HOXB7 as well as with AML risk groups. miR-196a-1 gene co-localizes with HOXB gene cluster between HOXB8 and HOXB13, positive correlation observed in AML patients between HOXB7 and mir-196a might be due to transcriptional co-regulation. miR-196a has been previously shown to be positively correlated with several HOX family members including HOXB7 and HOXA9 (Debernardi et al., 2007). In addition to miR-196a, we observed significant negative correlation between miR-16-HOXA10, miR-421-HOXA9, and miR-30e-HOXB7.

Among other miR target genes that were associated with worse OS were PIM-1 (pim1 oncogene), GZMB (granzyme B), and SEL1L3 (Sel-1 Suppressor Of Lin-12-Like 3). Pim1 is a serine/threonine protein kinase that has role in cell survival and cell proliferation. HOXA9 is transcriptional activator of Pim-1, which is further involved in regulation of MYC transcriptional activity, regulation of cell cycle progression, and phosphorylation and inhibition of proapoptotic proteins (BAD, MAP3K5, and FOXO3) thereby by contributing to its oncogenic activity. Pim-1 is also involved in inactivating MAP3K5 by phosphorylation thereby inhibiting MAP3K5-mediated phosphorylation of JNK and JNK/p38MAPK subsequently reducing caspase-3 activation and cell apoptosis. Pim-1 seems like a potential target for drug discovery, in fact in pediatric preclinical models, Pim1 inhibitor SGI-1776 has been shown to induce complete response to subcutaneous MV4:11 leukemia (Batra et al., 2012) as well as inhibit proliferation in other malignancies such as CLL, B cell lymphoma, multiple myeloma, etc. (Cervantes-Gomez et al., 2013, 2015; Yang et al., 2013).

GZMB was negatively regulated by miR-378 and higher expression was associated with worse OS in AML. GZMB is a key player in Granzyme signaling pathway, which is a lymphocyte granular serine protease that cleaves its substrates at Asp residues. GZMB is expressed in cytotoxic T lymphocytes (CTL) and NK cells and is primary mediator of apoptosis by CTL in cell-mediated immune response. GZMB seems to play critical role antibody –dependent cellular cytotoxicity (Elavazhagan et al., 2015).

Among miR target genes that were associated with good response were family members of Polypeptide *N*-Acetylgalactosaminyltransferases (GALNT1 and GALNT7), MAP4K4, TMEM87A, and COL3A1. GALNT1 was correlated negatively in expression with miR-10a, miR-16-2 and positively with miR-30e, which also demonstrated positive correlation with GALNT7 expression. MAP4K4 belongs to serine/threonine protein kinase family and has been specifically implicated in activation of MAPK8/JNK pathway.

Both MYB and TMEM87 were inversely associated with miR-107, which was associated with cytarabine resistance in cell lines as well as worse OS in AML patients (**Tables 2** and **3**). Oncogenic role of miR-107 in regulating tumor invasion and metastasis in gastric cancer by targeting DICER1 (Li et al., 2011) and in colorectal cancer by targeting metastasis suppressors death-associated protein kinase (DAPK) and Krüppel-like factor 4 (KLF4; Chen et al., 2012) has been proposed. In AML patients, we observed negative correlation of DICER with miR-107 (*p* < 0.01) although DICER expression was not predictive of outcome. Although TMEM87A a transmembrane protein has not been well studied, MYB, a V-Myb Avian Myeloblastosis Viral Oncogene Homolog has been implicated in leukemogenesis. Myb is reported to be overexpressed in AML and results from recent studies shows its potential role in interplay between C/EBPα activity for transcriptional regulation of FLT3 expression (Volpe et al., 2013). Recent report in luminal breast cancer demonstrated for the first time the potential tumor suppressor role of c-Myb gene, (Thorner et al., 2010), which is in concordance with TCGA results with Myb expression associated with better OS thereby warranting further investigation of Myb gene on its impact on treatment outcome in AML.

In summary, although several studies have established prognostic significance of miRNAs (miR-155, miR-29, miR-16, etc.) in AML, role of miRNAs in cytarabine chemosensitivity and development of resistance as a contributor of inferior outcome has not been well studied. In this report, we performed genome-wide miRNA profiling of eight AML cell lines and identified 20 miRNAs predictive of differential AML *in vitro* chemo-sensitivity (by measuring both cytarabine induced cell death and apoptosis). These were further investigated in AML patients using data from TCGA database, and in an integrated three way analyses of miRNA–target mRNA pairs with significant association and OS we identified 23 miRNA–mRNA-OS pairs of therapeutic importance in AML patients. Although additional functional validation studies to establish clinical/pharmacological importance of miRNA–mRNA pairs are needed, our preliminary data on RNA EMSAs confirmed binding of miR-107-MYB, miR10a-GALNT1, and miR-378-GZMB. Integration of pathogenic and pharmacologically significant miRNAs and miRNA–mRNA relationships opens up opportunities for development of targeted/miRNA-directed therapies.

## Author Contributions

JL, VL, and NB were involved in designing the study; NB, LC, and MS performed the experiments; NB, JL, XC, and SP performed statistical analysis. All authors contributed to manuscript writing. All authors read and approved the final manuscript.

## Conflict of Interest Statement

The authors declare that the research was conducted in the absence of any commercial or financial relationships that could be construed as a potential conflict of interest.

## Footnotes

1 cancergenome.nih.gov/

2 www.mirbase.org

## Supplementary Material

TABLE S1 **Association of MicroRNA with OS in AML Patients**.

FIGURE S1 **Characterization of acute myeloid leukemia (AML) Cell lines for Cytarabine chemosensitivity.** AML cell lines (*n* = 8) were cultured in the respective media and standard culturing conditions. **(A–H)** Cytarabine-induced cytotoxicity was determined by MTT assay after 48 h of drug treatment and area under the survival curve (AUC) was calculated using the cell survival data. Cytarabine-induced apoptosis was determined using caspase-3/7 assay after 48 h of drug treatment and relative caspase AUC was calculated.

FIGURE S2 **MicroRNA (miRNA) and cytarabine cell cytotoxicity.** Correlation plots showing association of selective miRNAs with cell survival AUC post cytarabine treatment of eight AML cell lines.

FIGURE S3 **Network of miRNAs and important biological processes.** miRNAs predictive of *in vitro* cytarabine chemosensitivity in cell lines as well as with overall survival (OS) in AML patients were analyzed using Ingenuity pathway analysis tools and pathways identified are depicted in the network.
