# Supplementary material for: MicroRNA–mRNA Pairs Associated with Outcome in AML: From In Vitro Cell-Based Studies to AML Patients
Source: Front Pharmacol. 2016 Jan 28;6:324. doi: 10.3389/fphar.2015.00324 (PMC4729948; doi:10.3389/fphar.2015.00324)
Supplement: TABLE S1 — Association of MicroRNA with OS in AML Patients. [file Table_1.PDF]

Supplementary Table 1: **Association of MicroRNA with OS in AML Patients.**

microRNA	miR-Risk group (P value)	miR-Risk group (BH95q)	miR-OS (Hazard Ratio)	miR-OS (P value)	miR-OS (BH95q)	miR-OS Risk stratified (HR)	miR-OS Risk stratified (P value)	miR-OS Risk stratified (BH95q)
hsa-mir-10a	**1.693E-12**	0.0000	1.1440	**0.0030**	0.0208	1.0860	0.1121	0.2615
hsa-mir-16	**2.163E-07**	0.0000	1.5620	**0.0114**	0.0481	1.1940	0.3617	0.5843
hsa-mir-196a	**7.793E-06**	0.0001	1.2210	**0.0029**	0.0208	1.1580	**0.0271**	0.1209
hsa-mir-197	**0.0004**	0.0019	1.3870	0.1687	0.2952	1.2030	0.4507	0.6761
hsa-mir-421	**0.0006**	0.0025	0.8890	0.2158	0.3211	0.9500	0.6267	0.7223
hsa-mir-155	**0.0130**	0.0391	1.4390	**0.0019**	0.0208	1.3120	**0.0288**	0.1209
hsa-mir-24-2	**0.0277**	0.0727	1.6250	**0.0181**	0.0544	1.4740	0.0801	0.2103
hsa-mir-374a	0.0829	0.1934	1.0020	0.9907	0.9907	1.1180	0.5264	0.6910
hsa-mir-148b	0.1040	0.2183	1.0660	0.7502	0.8753	1.2100	0.3570	0.5843
hsa-mir-107	0.1259	0.2305	1.5550	0.0966	0.2253	2.0430	**0.0085**	0.1209
hsa-mir-29a	0.1317	0.2305	1.2390	0.2294	0.3211	1.3770	0.0698	0.2095
hsa-mir-378	0.1697	0.2741	1.2470	0.1453	0.2849	1.2570	0.1622	0.3407
hsa-mir-324	0.2036	0.2850	1.1010	0.6380	0.7881	1.1550	0.5000	0.6910
hsa-mir-425	0.2269	0.2978	0.8670	0.2572	0.3375	0.9620	0.7708	0.7708
hsa-mir-30e	0.3016	0.3726	1.0350	0.9061	0.9703	0.9080	0.7395	0.7708
hsa-mir-29b	0.3613	0.4215	1.6630	**0.0077**	0.0407	1.5690	**0.0176**	0.1209
hsa-mir-25	0.4188	0.4481	0.6370	**0.0373**	0.0979	0.6210	**0.0359**	0.1258
hsa-mir-27b	0.7951	0.7951	0.7330	0.1950	0.3150	0.7370	0.1911	0.3648

P values < 0.05 are shaded and bold
