# Supplementary figures and images for: MicroRNA–mRNA Pairs Associated with Outcome in AML: From In Vitro Cell-Based Studies to AML Patients
Source: Front Pharmacol. 2016 Jan 28;6:324. doi: 10.3389/fphar.2015.00324 (PMC4729948; doi:10.3389/fphar.2015.00324)

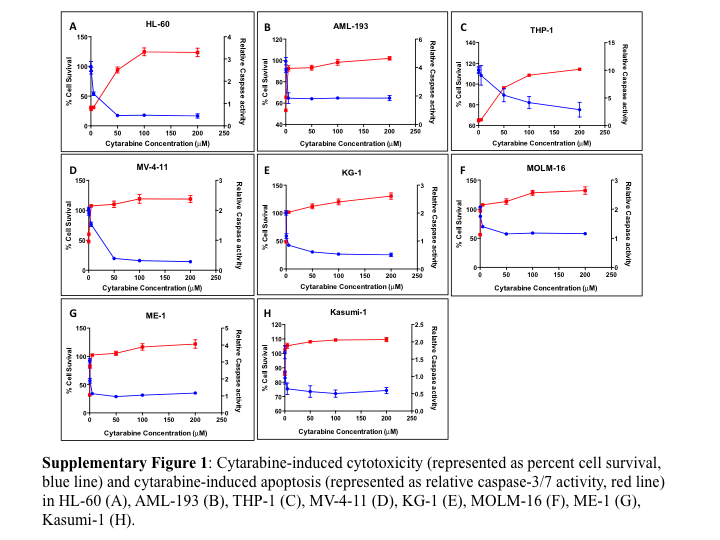

Supplement: FIGURE S1 — Characterization of acute myeloid leukemia (AML) Cell lines for Cytarabine chemosensitivity. AML cell lines (n = 8) were cultured in the respective media and standard culturing conditions. (A–H) Cytarabine-induced cytotoxicity was determined by MTT assay after 48 h of drug treatment and area under the survival curve (AUC) was calculated using the cell survival data. Cytarabine-induced apoptosis was determined using caspase-3/7 assay after 48 h of drug treatment and relative caspase AUC was calculated. [file Image_1.TIFF]

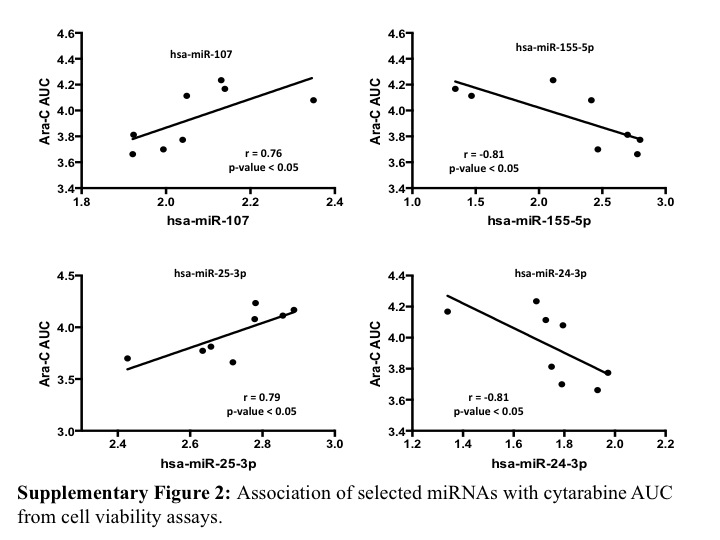

Supplement: FIGURE S2 — MicroRNA (miRNA) and cytarabine cell cytotoxicity. Correlation plots showing association of selective miRNAs with cell survival AUC post cytarabine treatment of eight AML cell lines. [file Image_2.JPEG]

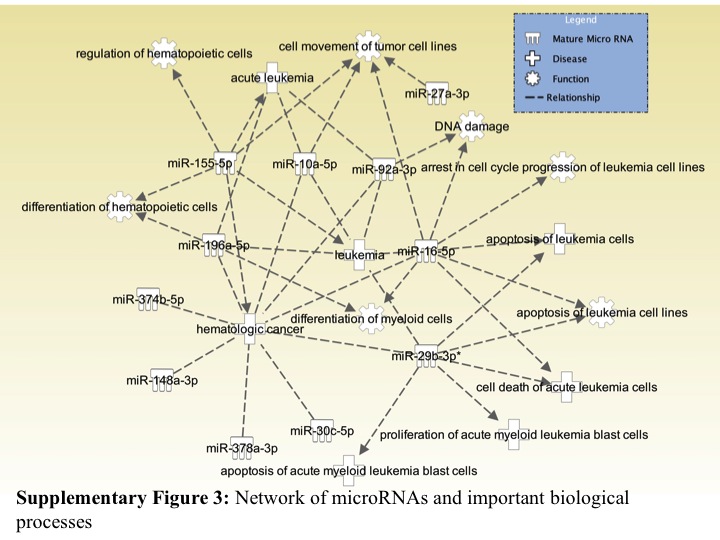

Supplement: FIGURE S3 — Network of miRNAs and important biological processes. miRNAs predictive of in vitro cytarabine chemosensitivity in cell lines as well as with overall survival (OS) in AML patients were analyzed using Ingenuity pathway analysis tools and pathways identified are depicted in the network. [file Image_3.JPEG]
